# Osteogenic Differentiation Capacity of In Vitro Cultured Human Skeletal Muscle for Expedited Bone Tissue Engineering

**DOI:** 10.1155/2017/8619385

**Published:** 2017-01-22

**Authors:** Chunlei Miao, Lulu Zhou, Lufeng Tian, Yingjie Zhang, Wei Zhang, Fanghong Yang, Tianyi Liu, Shengjian Tang, Fangjun Liu

**Affiliations:** ^1^Institute of Plastic and Reconstructive Surgery, Hospital for Plastic Surgery Affiliated to Weifang Medical University, Weifang, Shandong, China; ^2^Department of Orthopedic and Trauma Surgery, Weifang People's Hospital, Weifang, Shandong, China; ^3^Eugenom Inc., San Diego, CA 92121, USA; ^4^Department of Stomatology, Weifang People's Hospital, Weifang, Shandong, China; ^5^Department of Plastic and Cosmetic Surgery, Huadong Hospital Affiliated to Fudan University, Shanghai, China

## Abstract

Expedited bone tissue engineering employs the biological stimuli to harness the intrinsic regenerative potential of skeletal muscle to trigger the reparative process in situ to improve or replace biological functions. When genetically modified with adenovirus mediated BMP2 gene transfer, muscle biopsies from animals have demonstrated success in regenerating bone within rat bony defects. However, it is uncertain whether the human adult skeletal muscle displays an osteogenic potential in vitro when a suitable biological trigger is applied. In present study, human skeletal muscle cultured in a standard osteogenic medium supplemented with dexamethasone demonstrated significant increase in alkaline phosphatase activity approximately 24-fold over control at 2-week time point. More interestingly, measurement of mRNA levels revealed the dramatic results for osteoblast transcripts of alkaline phosphatase, bone sialoproteins, transcription factor CBFA1, collagen type I, and osteocalcin. Calcified mineral deposits were demonstrated on superficial layers of muscle discs after an extended 8-week osteogenic induction. Taken together, these are the first data supporting human skeletal muscle tissue as a promising potential target for expedited bone regeneration, which of the technologies is a valuable method for tissue repair, being not only effective but also inexpensive and clinically expeditious.

## 1. Introduction

Large segmental defects in craniofacial skeleton, particularly those secondary to ablative surgery, trauma, or congenital defects, do not heal well and continue to present clinical challenges to the surgeon [1, 2]. Although autogenous bone grafts and a wide range of biomaterials are routinely used for the facial bone repair, there are considerable disadvantages related with the current approaches, such as donor site morbidity, supply constraints, infection or rejection of the grafts, and high failure rate linked to the use of biomaterials [3–5]. Facilitated endogenous repair, an evolving discipline which avoids cell culture process and the use of manufactured scaffolds, has been proposed as a promising alternative to traditional tissue repair strategies within the field of craniofacial surgery [6–8]. This expedited tissue engineering strategy usefully employs biological stimuli to harness the intrinsic regenerative potential of endogenous tissues, such as muscle, fat, and bone marrow, to initiate the reparative processes in situ to improve or replace biological functions. Skeletal muscle has a propensity to form bone as seen most dramatically in the genetic disease fibrodysplasia ossificans progressiva (FOP) where patients develop a second skeleton as muscle spontaneously ossifies [9]. It is also well known that muscle contains progenitors cells that can be recruited in situ for osteogenesis and fragments of muscle provide the functions of a space-filling scaffold when implanted, making skeletal muscle an ideal candidate for this novel bone regeneration strategy [10]. More recently, skeletal muscle biopsies from animals, after being genetically modified with adenovirus mediated BMP2 gene transduction, have demonstrated success in new bone regeneration within rat critical size bony defects [7, 11]. Thus, the objective of this study was to investigate the high osteogenic differentiation potential of fresh human skeletal muscle tissue following in vitro cultivation with a standard osteogenic medium supplemented with dexamethasone, which is known to be an important regulator of mesenchymal progenitor cell differentiation.

## 2. Materials and Methods

### 2.1. Collection and Preparation of Skeletal Muscle

Under patients' informed consent, skeletal muscle biopsies were aseptically collected from 5 patients (3 males; 2 females) with an age range of 27–52 years, undergoing emergent treatment of open tibia fracture at the Department of Orthopedic and Trauma Surgery at Weifang Peoples' Hospital (Weifang, China). All procedures were followed by the surgical protocol preapproved by the institutional review board. Under aseptic conditions, all freshly harvested skeletal muscles (about 1.5 cm × 5 cm) were transferred to a laminar flow hood and washed for 5 times in phosphate-buffered saline containing 1% antibiotics. Afterwards, skeletal muscle was carefully removed from any residual fascia and tendon tissue and minced into approximately 2 mm thick slices in Petri dish. Standard 4 mm × 2 mm muscle discs were then produced with a 4 mm skin biopsy punch. These muscle discs were transferred to 6-well plates, and subsequently 1 mL alpha-MEM medium (supplemented with 10% fetal bovine serum and 1% antibiotics) was added to each well. The 6-well plate was placed back in the incubator (37°C; 5% CO_2_) and cultured overnight.

### 2.2. Osteogenic Differentiation of Skeletal Muscle

After preincubation for 24 hours, muscle discs were randomly distributed into two experimental groups, the osteogenic and control groups, in a 24-well plate. All induced groups were put on osteogenic medium supplemented with 10% fetal bovine serum plus 0.1 *μ*mol/L dexamethasone and 50 mg/L ascorbic acid, while control groups were cultured in alpha-MEM medium containing 10% fetal bovine serum alone. The plates were then placed in a humidified 37°C, 5% CO_2_ incubator and the differentiation medium was replenished every other day. After 2 weeks of incubation, part of muscle discs was collected and quantitatively assessed for alkaline phosphatase activity. All of the remaining muscle discs were continued to culture for an extended period of 8 weeks under the complete osteogenic medium containing 0.1 *μ*mol/L dexamethasone, 50 mg/L ascorbic acid, and 10 mmol/L beta-glycerol phosphate. All muscle discs were recovered and processed for RNA transcript measurements of bone matrix markers by quantitative, real-time PCR, or for histology by H&E and Alizarin Red stain.

### 2.3. Alkaline Phosphatase (ALP) Activity

The ALP activity was colorimetrically measured using an Alkaline Phosphatase Colorimetric Assay Kit (Sigma, St Louis, MO, USA), which uses p-nitrophenyl phosphate as phosphatase substrates. After exposure to basal osteogenic medium for 10 days, muscle discs were collected and washed twice with PBS solution. 250 *μ*L of phosphatase substrate solution was added to each well, and all samples were shielded from direct light for 10 min under 37°C, 5% CO_2_ incubator. Afterwards, same volume of 1 M sodium hydroxide was added to stop the reaction. 100 *μ*L final solution from each reaction was added to a 96-well plate for the absorbance to read at 405 nm on UVmax Microplate Reader (Manufacture, City, State). Alkaline phosphatase activity was expressed as nitrophenol product/minute using the standard curve and normalized to the total DNA content from each muscle disc that was analyzed as follows. All experiments were performed in triplicate.

### 2.4. DNA Content

Quant-iT™ PicoGreen® dsDNA Assay Kit (ThermoFisher Scientific, Grand Island, NY, USA) was used for measurement of genomic DNA extracted from each muscle disc. Closely following ALP enzyme reaction muscle discs were immediately washed twice with PBS, minced with a sterile scissor, and digested with 1 mg/mL collagenase type II (Sigma, USA) to release cells. After filtration and centrifugation, the supernatant was discarded and the cell pellet suspended with Tris-EDTA buffer was subjected to three cycles of freeze-thaw (at −80°C and 37°C, resp.) to release DNA. According to manufacturer's recommendation, cell lysate was mixed with the fluorescent dye and added to the wells of a black opaque 96-well plate. The fluorescence was then monitored with SpectraMax fluorometer at excitation/emission wavelengths of 485 nm/538 nm, respectively. All experiments were performed in triplicate.

### 2.5. Fluorescent Real-Time Polymerase Chain Reaction (FQ-PCR)

Individual muscle discs from each experimental group were frozen down in the liquid nitrogen at 2 weeks and 8 weeks' time points, respectively. Total RNA were extracted from homogenized samples in TRIzol® (ThermoFisher Scientific, Grand Island, NY), with a concentration quantified from the absorbance at 260 nm. 1 *μ*g total RNA was reverse transcribed into cDNA with Oligo-dt primers using an AMV RNA polymerase chain reaction kit (TaKaRa, Tokyo, Japan) and ready for FQ-PCR assay.

A highly sensitive fluorescent quantitative PCR method was performed on an ABI-7500 QPCR System (ThermoFisher Scientific, Grand Island, NY). The primers used for amplifying bone matrix marker genes are shown in Table 1 (designed and synthesized by Shanghai Sangon Biotech, China) [12]. The 20 *μ*L reaction mix contained 10 *μ*L 2 × SYBR® Premix TaqTM II (TaKaRa, Tokyo, Japan), 2 *μ*L template cDNA, and 0.4 *μ*M each primer. PCR cycles consisted of 10 min polymerase activation at 95°C followed by 40 amplification cycles: 95°C for 15 s and 55°C for 15 s. During expression analysis, each sample was amplified in triplicate. The average Ct value was calculated and a dissociation curve was generated by plotting each of the PCR products against its specific melting temperature (*Tm*) for verification. The levels of mRNA expression were normalized to those of the endogenous reference gene 18 S rRNA and reported as relative values (ΔΔCt) to those obtained from the control cells.

### 2.6. Matrix Mineralization

Muscle discs after 8-week in vitro osteogenic differentiation were collected and fixed in 4% paraformaldehyde for 2 hours. The specimens subsequently underwent dehydration in graded ethanol and clearance with serial xylene and then were embedded in paraffin. Serial 5 *μ*m paraffin sections were prepared and mounted on poly-L-lysine-coated slides and stained with 0.5% Alizarin Red S to detect any mineralized deposit in muscle discs.

### 2.7. Statistical Analysis

Comparisons of continuous variables between two treatment groups were performed using two-tailed Student's *t*-test with SPSS v11.5 software. Data are presented as means ± standard deviation (SD). The results were taken to be statistically significant at a probability level of *p* < 0.05. Unless otherwise noted, all experiments were run in triplicate and representative data are presented.

## 3. Results

### 3.1. Alkaline Phosphatase Enzyme Activity

**Figure 1:**
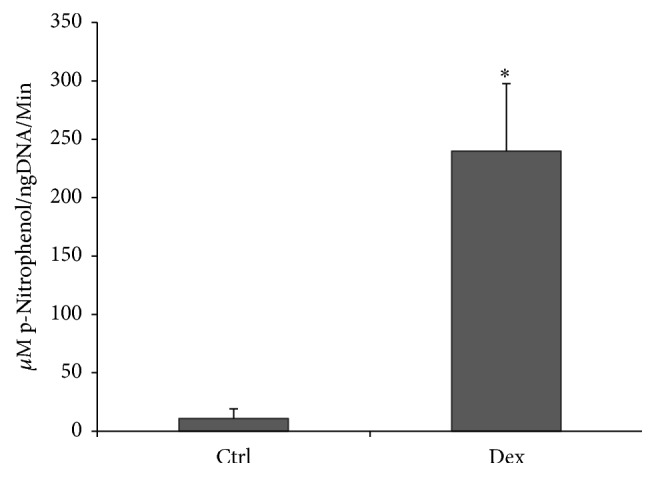
Detection of early marker for osteogenesis within induced skeletal muscle. Alkaline phosphatase enzyme activity within muscle discs was measured and normalized by total DNA contents for each muscle disc at 10 days after osteogenesis. *∗* denotes significant increase when compared to the control group (*p* < 0.05).

After 10 days in culture under osteogenic induction, alkaline phosphatase enzyme activity within muscle discs, an early marker of osteogenesis, increased approximately 24-fold over control groups (Figure 1), which is statistically significant between two groups.

### 3.2. Quantitative Reverse Transcription-Polymerase Chain Reaction

**Figure 2:**
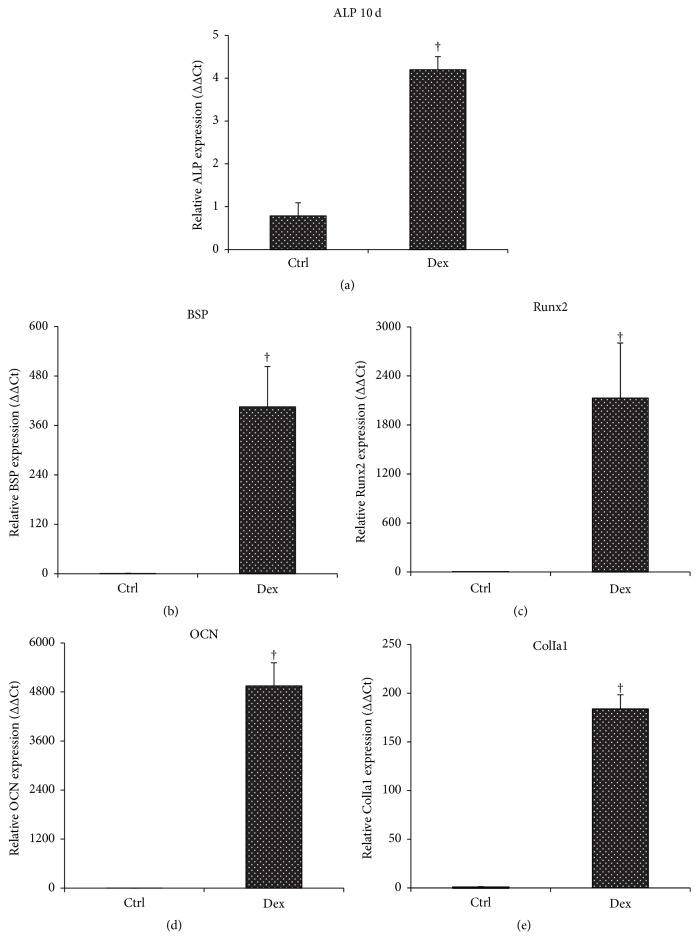
Expression of osteoblastic markers in muscle discs induced with osteogenic medium in vitro. Each muscle disc was extracted for total RNA and analyzed after 2 and 8 weeks of osteogenic induction, respectively, for expression of genes associated with osteogenesis by quantitative RT-PCR. † indicates significant difference (*p* < 0.05) relative to the control group. The experiments were performed in triplicate with muscle tissue harvested from 5 patients.

To gain insight into the possible increases for message expressions of bone matrix marker genes within muscle discs, quantitative real-time PCR measurements revealed that muscle discs induced with osteogenic medium showed a dramatic induction of transcripts associated with osteogenesis, including alkaline phosphatase (ALP), collagen type I, bone sialoprotein (BSP), transcription factor CBFA1 (Runx2), and osteocalcin (OCN). In consistency with ALP enzyme activity, the messenger RNA for ALP raised approximately five times the control levels by week 2 (Figure 2(a)). Meanwhile, transcript levels for relatively late osteogenic markers were also elevated significantly over controls, as shown in Figures 2(b), 2(c), 2(d), and 2(e). Data analysis indicates significant increases (*p* < 0.05) of induced muscle groups relative to control groups by two and eight weeks, respectively (Figure 2).

### 3.3. Calcium Deposition within Induced Muscle Discs

**Figure 3:**
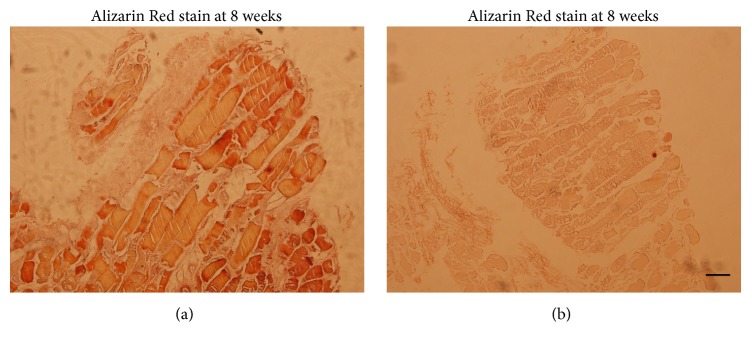
Osteogenic responses within human muscle discs cultured in vitro. Representative cross sections of Alizarin Red staining for muscle discs induced with osteogenic medium (a) or control discs (b) at 8 weeks were presented. The experiments were performed in triplicate with human skeletal muscle tissue harvested from 5 patients. Scale bar = 500 *μ*m.

Alizarin Red S is a dye that binds to calcium salts. When cultured for the extended period of 8 weeks in the presence of *β*-glycerol phosphate and ascorbic acid, muscle discs' cross sections in the induced groups demonstrated much more strong, distinct stain reaction with Alizarin Red dye, which revealed the deposition of calcified mineral on the superficial layer of in vitro cultured muscle discs (Figure 3(a)). No mineralized matrixes, in contrast, were noted within control muscle discs (Figure 3(b)).

## 4. Discussion

Bone tissue engineering strategies incorporate seed stem cells onto biocompatible and biodegradable scaffolds for regenerating or repairing musculoskeletal tissues, providing three elements required for new bone formation: osteoconduction, osteoinduction, and osteogenic cells [13, 14]. Despite relatively positive outcomes, bone tissue engineering has not proceeded to clinical practices due to several limitations or challenges. Multipotent mesenchymal stem cells have long been recognized for their potentials to regenerate bone grafts for the repair of bony defects [15–17]; however, limited cell population doublings and lack of knowledge about common markers for MSCs greatly hinder the actual amount and quality of MSCs obtainable for clinical application [18, 19]. In addition, some problems encountered with the use of either natural or synthetic materials for bone tissue engineering, such as scaffold porosity, degradation rate, poor biocompatibility, and mechanical integrity, remain largely unresolved [20, 21].

The need for a simple, expedited, and cost-effective technology for bone tissue engineering, which eventually avoids cell cultures and the use of manufactured scaffolds, is warranted. As an endogenous, natural, autologous scaffold provided by host tissue, the acellular muscle graft seeded with cultured Schwann cells for repairing the sciatic never defects showed a systematic and organized regeneration, including a proper orientation of regenerated axons [22, 23]. Meanwhile, several studies suggest the isolation of stem cells in skeletal muscle that is capable of differentiating into osteogenic lineages in vitro and accelerating healing of a skull defect in SCID mice in vivo [24, 25]. In response to this, Evans et al. from Harvard Medical School reported an alternative biologically based approach, called “facilitated endogenous repair,” for the rapid healing of large osseous and chondral defects, based upon the genetic modification of autologous skeletal muscle and fat grafts. These tissues were selected because they not only possess mesenchymal progenitor cells and scaffolding properties, but also can be biopsied, genetically modified, and returned to the patient in a single operative session [8]. This novel technique simplifies the traditional tissue engineering technology to a single, practical, cost-effective procedure without cell culture and the use of manufactured scaffolds and attempt to induce in situ tissue regeneration. After BMP-2 gene-activated muscle grafts implanting, rapid healing response of new bone and cartilage regeneration presented within rat critical-sized defects, with evidence of radiologic bridging and restoration of full mechanical strength [7, 11]. Liu et al. recently developed a practical translational model enabling the use of sheep skeletal muscle graft transduced with adenovirus expressing BMP2 for the repair of rat critical size femoral defects. The results confirmed the rapid and reliable healing of the defects in all rats and showed an early remodeling of newly regenerated bone tissue. By 8-week end point, vigorous remodeling finally reduced the presence of trabecular bone and led to well-organized new bone with advanced neocortication and rich bone marrow [26]. Thus far, these data demonstrate a strong theoretical basis for adapting this innovative technology for bone healing in large animal like sheep, as a prelude to future human trial.

Although the use of human muscle-derived progenitor cells for osteogenic differentiation and bone regeneration is well established [27, 28], no previous studies, to our knowledge, have reported direct in vitro induction of the intact human muscle tissue to undergo osteogenic differentiation while circumventing the drawbacks of cell growing and the use of bioscaffold in tissue engineering. Data from present study confirm the osteogenic activities in biopsies of human skeletal muscle when cultured in basal osteogenic medium, including the expression of bone matrix earlier marker of alkaline phosphatase and late bone markers of type I collagen, bone sialoprotein, and osteocalcin, and the visible deposition of extracellular mineral histologically on the superficial layer of induced muscle discs [29, 30]. Taken together, human skeletal muscle tissue, like those previously studied from other species [7, 11, 26], also displays the promising osteogenic differentiation potential in vitro when the biological trigger of dexamethasone, which is known to be an important regulator of mesenchymal progenitor cell differentiation, is applied.

Moreover, in considering available biological stimuli for use in current technology, more potent inducers for bone formation like bone morphogenetic proteins (BMPs), which are critical signaling molecules that instruct cells behavior during bone regeneration, have to be taken into consideration to this endeavor. Recombinant bone morphogenetic protein-2 (BMP-2) has been widely used as an effective growth factor in bone tissue engineering. After a prolonged phase of development, recombinant BMP2 products have become available for certain bone healing applications [8, 31–33]. However, relatively short half-life of proteins, problems associated with diffusion in the tissue, and large doses of required BMP-2 protein appear to limit its wide usefulness of direct delivery of growth factors in bone tissue engineering. It should also be pointed out that matrix-protein devices like InFuse bone grafts fail to deliver cells to the injury site [8].

From such considerations our future study is designed to induce skeletal muscle for osteogenesis in vitro based upon the genetic modification of muscle tissue using recombinant adenovirus vectors carrying human BMP-2 cDNA (Ad.BMP-2), a more practical and cost-effective strategy for osteogenic induction of muscle tissue. Ultimately, this could be implemented intraoperatively, with muscle biopsies harvested, genetically modified, and implanted into a defect in one session, which will most practically and cost-effectively merit the bone regeneration in an animal bony defect model and also future clinical applications [11, 26].

Overall, these data illustrate the advantages of using this expedited tissue repair approach to bone healing under different clinical settings. As suggested, skeletal muscle can be biopsied, triggered with suitable biological stimuli, and implanted within the framework of a single surgery without the need for expensive, time consuming ex vivo procedures in GMP (Good Manufacturing Practice) facilities [7]. In terms of its maneuverability and plasticity, skeletal muscle tissue graft used in this novel approach of facilitated endogenous repair can be molded to the contours of the lesion and also has good handling properties to facilitate its insertion into craniofacial bony defects, which is more variable in its size and shape and often accompanied by massive soft tissue loss due to the unique anatomical characteristics when compared to other parts of human skeleton. However, under these anatomical areas that are not surrounded by skeletal muscle, such as knee and cranial skull, muscle from another site, such as the latissimus dorsi or rectus abdominus, would be a better source [7, 8]. This could be harvested and transduced while other aspects of the surgery are taking place.

## Figures and Tables

**Table 1:** List and sequences of primers used for analysis of mRNA expression. All sequences are for human genes. F, forward; R, reverse.

Gene	Primer sequences (5′-3′)	Product sizes (bp)	Accession number	Primer efficiency (%)
18 S rRNA	F: CGGCTACCACATCCAAGGAA	187	NR 003286.2	98
	R: GCTGGAATTACCGCGGCT			
ALP	F: CGTGGCTAAGAATGTCATCATGTT	90	NM 000478.3	97
	R: TGGTGGAGCTGACCCTTGA			
Runx2	F: GCCTTCAAGGTGGTAGCCC	67	NM 001024630.2	96
	R: CGTTACCCGCCATGACAGTA			
COL1A1	F: TGGTTTCGACTTCAGCTTCC	92	NM 000088.3	95
	R: GAACCACATTGGCATCATCA			
OCN	F: GTAGTGAAGAGACCCAGGCG	99	NM 199173.2	97
	R: ATTGAGCTCACACACCTCCC			
BSP	F: GGCCTGTGCTTTCTCAATGAA	83	NM 004967.3	87
	R: GCCTGTACTTAAAGACCCCATTTTC			

ALP, alkaline phosphatase; Runx2, runt-related transcription factor 2; COL1A1, collagen type I, alpha 1 chain; OCN, osteocalcin; BSP, bone sialoprotein.
